# Evaluation of QuantiFERON-TB Gold for the Diagnosis of *Mycobacterium tuberculosis* Infection in HTLV-1-Infected Patients

**DOI:** 10.3390/v16121873

**Published:** 2024-11-30

**Authors:** Luana Leandro Gois, Natália Barbosa Carvalho, Fred Luciano Neves Santos, Carlos Gustavo Regis-Silva, Thainá Gonçalves Tolentino Figueiredo, Bernardo Galvão-Castro, Edgar Marcelino Carvalho, Maria Fernanda Rios Grassi

**Affiliations:** 1 Departamento de Biointeração, Instituto de Ciências da Saúde, Universidade Federal da Bahia, Salvador 40231-300, Bahia, Brazil; luana.leandro@ufba.br; 2 Laboratório Avançado de Saúde Pública, Instituto Gonçalo Moniz, Fundação Oswaldo Cruz (Fiocruz-BA), Salvador 40296-710, Bahia, Brazil; fred.santos@fiocruz.br (F.L.N.S.); carlos.regis@fiocruz.br (C.G.R.-S.); bgalvao@bahiana.edu.br (B.G.-C.); 3 Serviço de Imunologia, Hospital Universitário Professor Edgard Santos, Universidade Federal da Bahia, Salvador 40110-060, Bahia, Brazil; nataliacarvalho@ufba.br (N.B.C.); edgar.carvalho@fiocruz.br (E.M.C.); 4 Laboratório de Bacteriologia e Saúde, Instituto de Biologia, Universidade Federal da Bahia, Salvador 40170-110, Bahia, Brazil; 5 Escola Bahiana de Medicina e Saúde Pública, Salvador 40290-000, Bahia, Brazil; thainatolentino@hotmail.com; 6 Laboratório de Pesquisa Clínica, Instituto Gonçalo Moniz, Fundação Oswaldo Cruz (Fiocruz-BA), Salvador 40296-710, Bahia, Brazil

**Keywords:** HTLV-1, tuberculosis, tuberculin skin test, QuantiFERON

## Abstract

Human T-cell leukemia virus type 1 (HTLV-1) is associated with an increased risk of tuberculosis (TB). This study aimed to evaluate the performance of the QuantiFERON-TB Gold (QFT) test for the diagnosis of *Mycobacterium tuberculosis* (MTB) infection in HTLV-1-infected individuals. HTLV-1-infected participants were divided into four groups: HTLV-1-infected individuals with a history of tuberculosis (HTLV/TB), individuals with positive HTLV and tuberculin skin tests (HTLV/TST+) or negative TST (HTLV/TST−), and HTLV-1-negative individuals with positive TST results (HN/TST+). We compared the diagnostic performance of the QFT assay with that of the TST as a reference and evaluated test sensitivity, specificity, accuracy, likelihood ratio, and diagnostic odds ratio. The results showed a higher frequency of positive TST results and induration diameter ≥10 mm in HTLV-1-infected individuals than in the controls. The QFT test was more frequently positive in the HTLV/TB group than in the other groups, while a combined analysis of HTLV/TB and HTLV/TST+ indicated a QFT sensitivity of 57.5%. No significant differences were found in the other diagnostic performance measures, as QFT test results were in agreement with TST results, particularly in TST-negative individuals. Given the low sensitivity of QFT for LTBI in individuals infected with HTLV-1, the TST may be preferable in regions where both infections are endemic.

## 1. Introduction

Human T-cell leukemia virus type 1 (HTLV-1) and *Mycobacterium tuberculosis* (MTB) both constitute major public health problems in Brazil. There is a paucity of data on the prevalence of HTLV-1 infection in the general population; however, it is feasible to estimate this rate based on serological screening of blood donors, pregnant women, and other selected population groups [1]. HTLV-1 affects an estimated 5–10 million individuals globally, with a notably high prevalence in Central and South America, Central Africa, and southwestern Japan. Brazil is home to around 800,000 HTLV-1-infected persons, representing one of the largest endemic areas worldwide for diseases associated with HTLV-1 [2]. Bahia, a state located in Northeast Brazil, is considered the epicenter of this infection. A study evaluating samples from the Bahia Central Laboratory of Public Health found an estimated prevalence of 0.84%, corresponding to approximately 130,000 infected individuals in this state [3]. Moreover, it is estimated that 10.6 million people worldwide contracted tuberculosis in 2022, and approximately 1.3 million deaths among HIV-negative people are attributed annually to tuberculosis [4]. In Brazil, tuberculosis remains a significant public health concern, especially in the city of Salvador, where around 3000 new cases of tuberculosis are reported every year [4].

Epidemiologic studies in Brazil have shown a significant correlation between HTLV-1 and MTB co-infection, with higher rates of HTLV-1 prevalence noted in TB patients in Bahia (4.27–11.1%) compared to the Brazilian general population (1.32–1.8%) [5,6,7,8]. HTLV-1 infection has been observed to triple the incidence of TB, especially in people aged 31 to 50 years [9]. Patients with HTLV-1 infection present a reduced in vitro response to MTB antigens, as well as decreased cellular proliferation [10] and cytokine production [11]. In addition, a study conducted in Japan found that HTLV-1-infected carriers showed a weaker response to the tuberculin skin test (TST) compared to healthy controls [12].

To diagnose latent tuberculosis infection (LTBI), healthcare professionals typically use the tuberculin skin test (TST) and interferon-gamma (IFN-γ) release assays (IGRAs), including T-SPOT.TB and QuantiFERON-TB Gold (QFT). Since only a few studies have evaluated the specificity of IGRA in the context of HTLV-1, it is important to evaluate test performance due to the potential for altered cytokine responses in HTLV-1-infected individuals. Accordingly, this study aimed to assess the capacity of the QFT method to diagnose MTB infection in HTLV-1-infected patients, considering their unique immunologic profiles.

## 2. Materials and Methods

### 2.1. Population

This cross-sectional study included 162 HTLV-1-infected individuals recruited from the Integrated and Multidisciplinary HTLV Center (CHTLV) at the Bahiana School of Medicine and Public Health (EBMSP) and the HTLV-1 Clinic at the Professor Edgar Santos University Hospital Complex, Federal University of Bahia (HUPES-UFBA), both located in Salvador, Northeast Brazil. Eligible participants were 18 years or older, tested positive for HLTV-1 by ELISA and Western blot, and were classified as asymptomatic according to the De Castro-Costa criteria [13]. Individuals with positive serology for HIV, HBV or HCV, or those undergoing corticosteroid therapy, were excluded from the study. The control group consisted of 162 uninfected individuals (HTLV-1-negative or HNs) from these same institutions. This study was approved by the Institutional Research Board of EBMSP (Protocol Nº029/2010), and all participants provided written informed consent.

### 2.2. Tuberculin Skin Test

Tuberculin skin testing was performed in all study participants. The diameter of induration was measured 48 h after injection of the purified protein derivative (PPD). Induration reactions of 5–9 mm were classified as weak responses, while those over 9 mm were classified as strong. Indurations under 5 mm were classified as non-reactive.

### 2.3. Definition of Tuberculosis Case

A tuberculosis case (TB) was defined as a patient presenting with respiratory symptoms and a positive smear for MTB. Diagnosis could also be confirmed based on positive culture results, or a clinical history of tuberculosis supported by tests such as chest radiographs indicating pulmonary abnormalities [14].

### 2.4. Interferon-γ Release Assay

Blood samples from 66 HTLV-infected participants and 24 HNs were analyzed using the QuantiFERON-TB Gold (QFT; QIAGEN, Germantown, MD, USA) assay. Participants were classified as infected with MTB if differences in IFN-γ levels between cells stimulated with specific antigens and non-stimulated cells were greater than 0.35 IU/mL, and at least 25% higher than unstimulated cells. Negative results were defined by differences of less than 0.35 IU/mL, or an increase in IFN-γ production of less than 25%. Indeterminate results were identified by specific criteria, including inadequate responses to non-specific stimulators.

### 2.5. HTLV-1 Proviral Load

The HTLV-1 proviral load was quantified by real-time TaqMan PCR as described elsewhere [15].

### 2.6. Statistical Analysis

**Figure 1:**
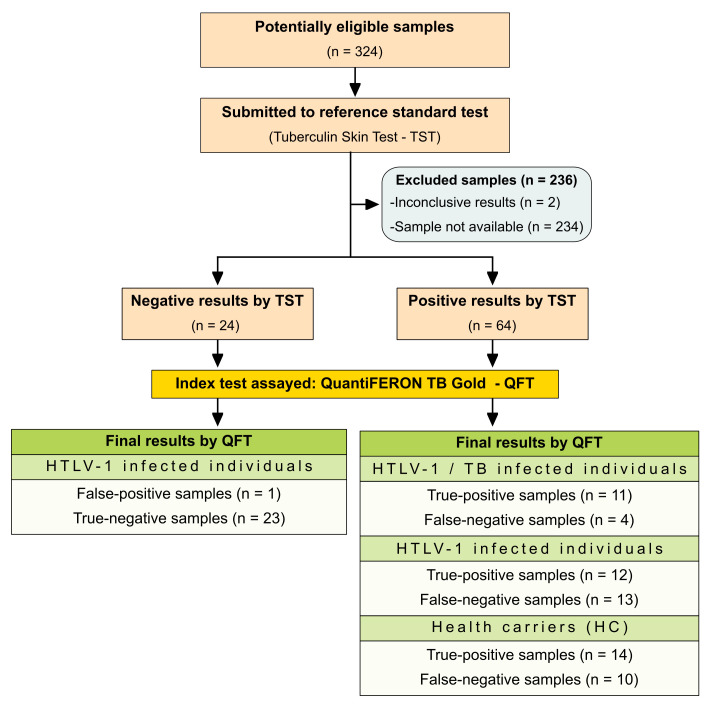
Flowchart illustrating study design in conformity with the Standards for Reporting of Diagnostic Accuracy Studies (STARD).

Data are presented as medians and interquartile ranges. The Kruskal–Wallis test was used to evaluate differences between median values in groups, followed by Dunn’s post-test for pairwise comparisons. The chi-square test assessed differences in distribution frequencies. GraphPad Prism software version 9.5.1 (GraphPad Inc., San Diego, CA, USA) supported our statistical analysis. The diagnostic performance of the QFT assay was assessed in terms of sensitivity, specificity, accuracy, likelihood ratio (LR) and diagnostic odds ratio (DOR), with TST serving as the reference standard. Confidence intervals (CIs) were calculated at 95% and *p*-values < 0.05 were considered statistically significant. The absence of overlapping 95% CI values was deemed indicative of statistical significance. The strength of agreement between TST and QFT results was evaluated by Cohen’s Kappa coefficient (k), interpreted as follows: no agreement (k = 0), slight agreement (0.20 ≤ k > 0), fair agreement (0.40 ≤ k ≥ 0.21), moderate agreement (0.60 ≤ k ≥ 0.41), substantial agreement (0.80 ≤ k ≥ 0.61), and almost perfect agreement (1.0 ≤ k ≥ 0.81) (Landis and Koch 1977). This study adhered to the STARD guidelines for diagnostic accuracy reporting [16], and a detailed checklist and flowchart (Figure 1) is provided to ensure transparency and replicability.

## 3. Results

TST reactivity was examined in 162 HTLV-1-infected individuals and 162 HNs. There were no significant age differences between the HTLV-1-infected and uninfected groups, yet a higher proportion of women was observed in the HTLV-1-infected group compared to HNs. TST positivity was significantly higher in the HTLV-1-infected individuals (53.7%) than in HNs (34.6%; *p* = 0.001). In addition, a greater proportion of HTLV-1-infected individuals (42%) presented an induration diameter of 10 mm or more compared with 29% of HNs (*p* = 0.0015), as shown in Table 1.

In total, 90 participants (63 women and 27 men) were categorized into four groups based on TST results, history of tuberculosis, and HTLV-1 infection status: HTLV-1-infected individuals with a history of tuberculosis (HTLV/TB; *n* = 16), HTLV-1-infected individuals with a positive TST (HTLV/TST+; *n* = 25), HTLV-1-infected individuals with a negative TST (HTLV/TST-; *n* = 25), and HNs with a positive TST (HNs/TST+; *n* = 24). Two individuals with indeterminate QFT results were excluded from the analysis, one from the HTLV/TB group and one from the HTLV/TST- group. HTLV-1-infected individuals were found to be older on average compared to the HNs/TST+ group (*p* = 0.0002), as shown in Table 2. The median induration diameter and HTLV-1 proviral load showed no significant differences between the groups.

QFT positivity rates varied between groups, as shown in Table 3. The HTLV/TB group had the highest QFT positivity rate at 71.4%, followed by HN/TST+ with 58.3%, HTLV/TST+ with 48.0%, and HTLV/TST− with 4.2%.

**Figure 2:**
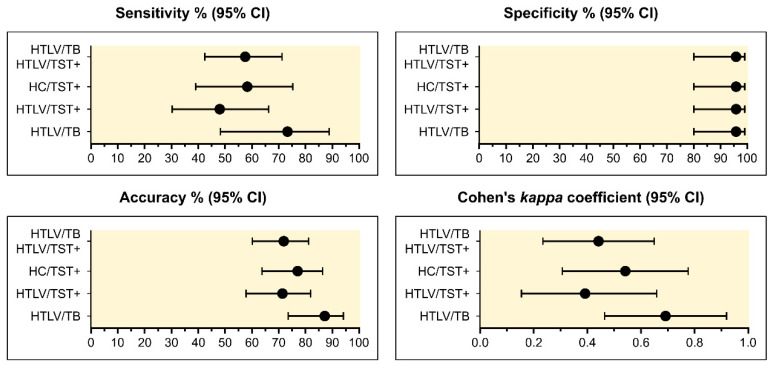
Diagnostic parameters used to evaluate the performance of the QuantiFERON-TB (QFT) assay in detecting TST positivity and previous tuberculosis infection (TB) among individuals with HTLV-1. HN (HTLV-1-negative); HTLV (HTLV-1-infected individuals); TB (tuberculosis); TST (tuberculin skin test).

Evaluation of the QFT assay’s diagnostic performance revealed no significant differences in sensitivity among the MTB-infected groups (HN/TST+, HTLV/TST+, and HTLV/TB), as shown in Figure 2. Specifically, the sensitivity rates were 58.3% (95% CI: 38.8–75.5%) for HC/TST+, 48.0% (95% CI: 30.0–66.5%) for HN/TST+, and 73.3% (95% CI: 48.0–89.1%) for HTLV/TB.

Our findings suggest that HTLV-1 infection does not contribute to false-negative QFT results. A combined analysis focusing on both MTB infection and latent TB (LTB) in HTLV-1-infected participants (HTLV/TB and HTLV/TST+ groups) was used to calculate QFT sensitivity, while data from HTLV/TST- participants were used to determine QFT specificity. The overall results indicated a QFT sensitivity of 57.5% (95% CI: 42.2–71.5%), a specificity of 95.8% (95% CI: 79.8–99.3%), and an accuracy of 71.9% (95% CI: 59.9–81.4%).

Cohen’s Kappa values indicated substantial agreement between QFT and TST in the HTLV/TB group (k = 0.79; 95% CI: 0.49–0.95) and moderate agreement in HN/TST+ (k = 0.54; 95% CI: 0.30–0.78), HTLV/TST+ (k = 0.43; 95% CI: 0.18–0.69), and combined HTLV/TB and HTLV/TST+ groups (k = 0.47; 95% CI: 0.26–0.68). The diagnostic odds ratio (DOR) showed variation across the groups. The highest DOR value was observed in the HTLV/TB group at 63.3 (95% CI: 6.3–634.8), followed by HN/TST+ at 32.2 (95% CI: 3.7–279.3), combined HTLV/TB and HTLV/TST+ groups at 31.1 (95% CI: 3.8–253.6), and HTLV/TST+ alone at 21.2 (95% CI: 2.5–182.3). Despite variability in DOR values, the overlapping 95% CI across the different metrics (sensitivity, specificity, accuracy, and Cohen’s Kappa coefficient) indicates no significant statistical differences in the QFT test performance among the analyzed groups.

## 4. Discussion

This study examined the performance of the QFT test in diagnosing MTB infection in HTLV-1-infected individuals compared to HNs. Our results show that, despite a higher frequency of positive TST results among the HTLV-1-infected groups, QFT positivity was notably lower in HTLV-1-infected individuals with a positive TST (HTLV/TST+) compared to HN/TST+ and HTLV/TB. In addition, the present study identified concordance between TST and QFT results, as assessed by Cohen’s Kappa coefficient, with mostly moderate agreement observed between groups. QFT performance was not found to be statistically significant between groups due to overlapping 95% CIs.

The diagnostic value of IGRAs for LTBI may vary depending on the population under study. IGRAs have been reported to be more effective than TST in diagnosing active TB infection or LTBI in the general population [17]. In immunocompetent individuals exposed to patients with active pulmonary TB, a study reported approximately 77% accuracy using QFT compared to TST [18]. However, other studies have found higher rates of positivity with QFT (70.3%) compared to TST (52.5%) [19,20]. Few studies have examined IGRAs in HTLV-1-infected populations, with contradictory results. A study in Peru found moderate agreement between QTF and TST in diagnosing latent MTB in HTLV-1-infected patients, similar to the results of the present study [21]. In contrast, another study found that approximately 55% of HTLV-1-infected patients with rheumatoid arthritis had invalid T-SPOT.TB results due to high background activity [22]. In the present study, indeterminate results by QTF were only identified in two HTLV-1-infected individuals (one with a past history of TB and another with TST- result), indicating that diagnosis method may potentially offer decreased sensitivity in HTLV-1-infected individuals. Although not statistically significant, the frequency of QTF positivity was slightly lower in the HTLV/TST+ group than that of uninfected TST+ individuals (HNs) in the present study. The lower QFT response seen in HTLV/TST+ compared to HNs may be related to the immunologic changes induced by HTLV-1. This virus primarily infects CD4+ T cells, leading to chronic infection by integrating its genome into host DNA, triggering spontaneous proliferation of immune cells and secretion of inflammatory cytokines, including TNF, IFN-γ, and IL-6 [23,24,25,26,27]. HTLV-1 infection has been shown to alter the immune response to MTB antigens, resulting in reduced proliferation [10] and cytokine production, notably in TNF, IL-17 and IL-1β [11]. Additionally, patients with HTLV-1-associated myelopathy and tuberculosis exhibit higher TNF/IL-10 and IFN-γ/IL-10 ratios compared to those without TB [28].

Interestingly, the proportion of HTLV-1-infected individuals with a positive TST was similar to that of HNs in the present study, with no differences in TST induration size observed. This stands in contrast to a Japanese study that reported a lower frequency of positive TST reactions in HTLV-1-infected individuals [12]. These discordant results may be partially explained by epidemiological differences between the two countries. First, Brazil has a higher burden of *M. tuberculosis* infection than Japan, as Brazil is on the WHO’s list of 30 high TB burden countries, accounting for 87% of global TB cases [4]. In addition, the lower response to TST described in HTLV-1-infected individuals in Japan, as evidenced by a lack/reduced size of induration in response to TB antigens, was reported mainly in older HTLV-1 carriers (>60 years) [12]. In the present study, the mean age of HTLV-1-infected individuals tested for TST was less than 60 years.

A relevant limitation of this study is the reliance on positive TST as the gold standard for latent MTB infection. Standard TB diagnostic methods, such as sputum smear microscopy and molecular testing, are not applicable for latent infection. Additionally, we did not include HTLV-1-uninfected individuals with negative TST as controls, nor did we evaluate IFN-γ production by T-SPOT.TB, which could provide additional information on MTB infection. Another limitation was the small sample size of individuals evaluated by QTF, which limited the capability of statistical analyses.

## 5. Conclusions

In conclusion, our results demonstrate moderate agreement between QFT and TST in the diagnosis of latent MTB infection. However, considering the low sensitivity of QTF in the diagnosis of LTBI in HTLV-1-infected individuals, TST may be preferred option in areas for where both infections are endemic. Larger studies across different regions with variable HTLV-1 and TB prevalence are needed to confirm and generalize these findings.

## Figures and Tables

**Table 1:** Tuberculin skin test positivity and demographic characteristics of HTLV-1-infected subjects and healthy controls.

	HTLV (*n* = 162)	HNs (*n* = 162)	*p*-Value *
Age (years)	53.2 ± 12.3	46.6 ± 15.6	>0.05
Females, n (%)	111 (68.5%)	82 (50.6%)	0.001
TST negative	75 (46.3%)	106 (65.4%)	0.001
TST positive	87 (53.7%)	56 (34.6%)	0.001
5 ≥ ID < 10	19 (11.7%)	9 (5.6%)	0.048
ID > 10 mm	68 (42.0%)	47 (29.0%)	0.015

HNs (HTLV-1-negative); HTLV (HTLV-1-infected individuals); ID (induration diameter); TST (tuberculin skin test). * Chi-squared test.

**Table 2:** Demographic characteristics, TST, and proviral load in study groups.

	HTLV/TB (*n* = 15)	HTLV/TST+ (*n* = 25)	HTLV/TST− (*n* = 24)	HN/TST+ (*n* = 24)	*p*-Value *
Age (years)	65 (55–72)	51 (46–61)	51 (41–63)	29 (27–43)	0.0002
Females, n (%)	11 (69%)	14 (56%)	20 (80%)	18 (75%)	0.3
TST (mm)	19.5 (16–22)	14 (10–20)	<5	16 (15–20)	0.17
PVL (copies/106)	25,111 (416–59,926)	10,264 (845–48,668)	6266 (332–52,160)	NP	0.8

Data are presented as medians (interquartile range). HN (HTLV-1-negative); HTLV (HTLV-1-infected individuals); NP (not performed); PVL (proviral load); TB (tuberculosis); TST (tuberculin skin test). * Chi-squared test (age, TST, and PL) and Kruskal–Wallis (females).

**Table 3:** QuantiFERON-TB (QFT) assay performed in four different analyses groups.

QTF Results * n (%)	HTLV/TB (*n* = 15)	HTLV/TST+ (*n* = 25)	HTLV/TST− (*n* = 24)	HN/TST+ (*n* = 24)
Positive	10 (71.4)	12 (48.0)	1 (4.2)	14 (58.3)
Negative	4 (26.7)	13 (52.0)	23 (95.8)	10 (41.7)

* Indeterminate results from the QuantiFERON-TB (QFT) assay were excluded. HC (healthy controls); HTLV (HTLV-1-infected individuals); TB (tuberculosis); TST (tuberculin skin test).

## Data Availability

The data presented in this study are available on request from the corresponding author due to restrictions on patient information.

## References

[1] Hlela C., Shepperd S., Khumalo N.P., Taylor G.P. (2009). The Prevalence of Human T-Cell Lymphotropic Virus Type 1 in the General Population Is Unknown. AIDS Rev..

[2] Gessain A., Cassar O. (2012). Epidemiological Aspects and World Distribution of HTLV-1 Infection. Front. Microbiol..

[3] Pereira F.M., de Almeida M.d.C.C., Santos F.L.N., Carreiro R.P., Regis-Silva C.G., Galvão-Castro B., Grassi M.F.R. (2019). Evidence of New Endemic Clusters of Human T-Cell Leukemia Virus (HTLV) Infection in Bahia, Brazil. Front. Microbiol..

[4] Bagcchi S. (2023). WHO’s Global Tuberculosis Report 2022. Lancet Microbe.

[5] Moreira E.D., Ribeiro T.T., Swanson P., Sampaio Filho C., Melo A., Brites C., Badaró R., Toedter G., Lee H., Harrington W. (1993). Seroepidemiology of Human T-Cell Lymphotropic Virus Type I/II in Northeastern Brazil. J. Acquir. Immune Defic. Syndr..

[6] Pedral-Sampaio D., Martins Netto E., Pedrosa C., Brites C., Duarte M., Harrington W. (1997). Co-Infection of Tuberculosis and HIV/HTLV Retroviruses: Frequency and Prognosis Among Patients Admitted in a Brazilian Hospital. Braz. J. Infect. Dis..

[7] de Lourdes Bastos M., Osterbauer B., Mesquita D.L., Carrera C.A., Albuquerque M.J., Silva L., Pereira D.N., Riley L., Carvalho E.M. (2009). Prevalence of Human T-Cell Lymphotropic Virus Type 1 Infection in Hospitalized Patients with Tuberculosis. Int. J. Tuberc. Lung Dis..

[8] Marinho J., Galvão-Castro B., Rodrigues L.C., Barreto M.L. (2005). Increased Risk of Tuberculosis with Human T-Lymphotropic Virus-1 Infection: A Case-Control Study. J. Acquir. Immune Defic. Syndr..

[9] Grassi M.F.R., Dos Santos N.P., Lírio M., Kritski A.L., Chagas Almeida M.d.C., Santana L.P., Lázaro N., Dias J., Netto E.M., Galvão-Castro B. (2016). Tuberculosis Incidence in a Cohort of Individuals Infected with Human T-Lymphotropic Virus Type 1 (HTLV-1) in Salvador, Brazil. BMC Infect. Dis..

[10] Mascarenhas R.E., Brodskyn C., Barbosa G., Clarêncio J., Andrade-Filho A.S., Figueiroa F., Galvão-Castro B., Grassi F. (2006). Peripheral Blood Mononuclear Cells from Individuals Infected with Human T-Cell Lymphotropic Virus Type 1 Have a Reduced Capacity to Respond to Recall Antigens. Clin. Vaccine Immunol..

[11] Carvalho N.B., de Lourdes Bastos M., Souza A.S., Netto E.M., Arruda S., Santos S.B., Carvalho E.M. (2018). Impaired TNF, IL-1β, and IL-17 Production and Increased Susceptibility to Mycobacterium Tuberculosis Infection in HTLV-1 Infected Individuals. Tuberculosis.

[12] Tachibana N., Okayama A., Ishizaki J., Yokota T., Shishime E., Murai K., Shioiri S., Tsuda K., Essex M., Mueller N. (1988). Suppression of Tuberculin Skin Reaction in Healthy HTLV-I Carriers from Japan. Int. J. Cancer.

[13] Castro-Costa C.M.D., Araújo A.Q.C., Barreto M.M., Takayanagui O.M., Sohler M.P., Silva E.L.M.D., Paula S.M.B.D., Ishak R., Ribas J.G.R., Rovirosa L.C. (2006). Proposal for Diagnostic Criteria of Tropical Spastic Paraparesis/HTLV-I-Associated Myelopathy (TSP/HAM). AIDS Res. Hum. Retroviruses.

[14] Castelo Filho A., Kritski A.L., Barreto Â.W., Lemos A.C.M., Netto A.R., Guimarães C.A., Silva C.L., Sant’anna C.d.C., Haddad D.J., Lima D.S. (2004). II Consenso Brasileiro de Tuberculose: Diretrizes Brasileiras para Tuberculose 2004. J. Bras. Pneumol..

[15] Dehée A., Césaire R., Désiré N., Lézin A., Bourdonné O., Béra O., Plumelle Y., Smadja D., Nicolas J.C. (2002). Quantitation of HTLV-I Proviral Load by a TaqMan Real-Time PCR Assay. J. Virol. Methods.

[16] Cohen J.F., Korevaar D.A., Altman D.G., Bruns D.E., Gatsonis C.A., Hooft L., Irwig L., Levine D., Reitsma J.B., de Vet H.C.W. (2016). STARD 2015 Guidelines for Reporting Diagnostic Accuracy Studies: Explanation and Elaboration. BMJ Open.

[17] Pai M., Riley L.W., Colford J.M. (2004). Interferon-Gamma Assays in the Immunodiagnosis of Tuberculosis: A Systematic Review. Lancet Infect. Dis..

[18] Ferreira T.F., Matsuoka P.d.F.S., Santos A.M.D., Caldas A.d.J.M. (2015). Diagnosis of Latent Mycobacterium Tuberculosis Infection: Tuberculin Test versus Interferon-Gamma Release. Rev. Soc. Bras. Med. Trop..

[19] Conde M.B., de Melo F.A.F., Marques A.M.C., Cardoso N.C., Pinheiro V.G.F., Dalcin P.d.T.R., Machado Junior A., Lemos A.C.M., Netto A.R., Durovni B. (2009). III Diretrizes para Tuberculose da Sociedade Brasileira de Pneumologia e Tisiologia. J. Bras. Pneumol..

[20] Takenami I., Loureiro C., Machado A., Emodi K., Riley L.W., Arruda S. (2013). Blood Cells and Interferon-Gamma Levels Correlation in Latent Tuberculosis Infection. ISRN Pulmonol..

[21] Luhmann K., Vasquez N., Hoces D., Gonzalez E., Brewer T., Gotuzzo E., Montes M. (2016). Interferon Gamma Release Assay and Tuberculin Skin Test Agreement for the Diagnosis of Latent Tuberculosis in Human T- Lymphotropic Virus Subjects. Open Forum Infect. Dis..

[22] Umekita K., Hashiba Y., Iwao K., Iwao C., Kimura M., Kariya Y., Kubo K., Miyauchi S., Kudou R., Rikitake Y. (2020). Human T-Cell Leukemia Virus Type 1 May Invalidate T-SPOT.TB Assay Results in Rheumatoid Arthritis Patients: A Retrospective Case-Control Observational Study. PLoS ONE.

[23] Etoh K., Tamiya S., Yamaguchi K., Okayama A., Tsubouchi H., Ideta T., Mueller N., Takatsuki K., Matsuoka M. (1997). Persistent Clonal Proliferation of Human T-Lymphotropic Virus Type I-Infected Cells in Vivo. Cancer Res..

[24] Santos S.B., Porto A.F., Muniz A.L., Luna T., Nascimento M.C., Guerreiro J.B., Oliveira-Filho J., Morgan D.J., Carvalho E.M. (2006). Modulation of T Cell Responses in HTLV-1 Carriers and in Patients with Myelopathy Associated with HTLV-1. Neuroimmunomodulation.

[25] Olah I., Fukumori L.M.I., Montanheiro P., Vergara M.P., Smid J., Duarte A.J.S., Penalva de Oliveira A.C., Casseb J. (2007). Patterns of in Vitro Lymphoproliferative Responses among HTLV-1-Infected Subjects: Upregulation by HTLV-1 during HIV-1 Co-Infection. Scand. J. Immunol..

[26] Futsch N., Prates G., Mahieux R., Casseb J., Dutartre H. (2018). Cytokine Networks Dysregulation during HTLV-1 Infection and Associated Diseases. Viruses.

[27] Ahuja J., Lepoutre V., Wigdahl B., Khan Z.K., Jain P. (2007). Induction of Pro-Inflammatory Cytokines by Human T-Cell Leukemia Virus Type-1 Tax Protein as Determined by Multiplexed Cytokine Protein Array Analyses of Human Dendritic Cells. Biomed. Pharmacother..

[28] Souza A., Carvalho N., Neves Y., Braga Santos S., Bastos M.d.L., Arruda S., Netto E.M., Glesby M.J., Carvalho E. (2017). Association of Tuberculosis Status with Neurologic Disease and Immune Response in HTLV-1 Infection. AIDS Res. Hum. Retroviruses.

